# Are age and anti-Müllerian hormone good predictors of ovarian reserve and response in women undergoing IVF?

**DOI:** 10.5935/1518-0557.20180043

**Published:** 2018

**Authors:** Juliano Augusto Brum Scheffer, Bruno Scheffer, Rafaela Scheffer, Fabio Florencio, Michael Grynberg, Daniel Mendez Lozano

**Affiliations:** 1 IBRRA – Brazilian Institute of Assisted Reproduction, Belo Horizonte, Brazil; 2 Department of Reproductive Medicine, Hôpital Jean Verdier (AP-HP), University Paris XIII, and INSERM, Paris, France; 3 School of Medicine, Tecnologico de Monterrey and Center for Reproductive Medicine CREASIS, San Pedro Monterrey, Mexico

**Keywords:** Age, anti-Müllerian hormone, antral follicle count, ongoing pregnancy

## Abstract

**Objective:**

Ovarian reserve evaluation has been the focus of substantial clinical research for several years. This study aimed to examine the associations between markers of ovarian reserve and ovarian response.

**Methods:**

This prospective study included 132 infertile women aged 24-48 years undergoing routine exploration during unstimulated cycles prior to the start of assisted reproductive technology (ART) treatments at our center from July 2015 to January 2017. Descriptive parameters and patient characteristics were reported as mean (SD) or median (range) values depending on the data distribution pattern. Student’s t-test was performed for continuous variables; the Wilcoxon and Pearson’s test were used for data not following a normal distribution; and Fisher’s test was used for categorical variables. *p*<0.05 was considered statistically significant.

**Results:**

At the time of the study, the patients had a mean age of 35.7±3.84 years. On day 3 of the cycle, the mean anti-Müllerian hormone (AMH) serum level was 2.84±1.57 ng/mL and the patients had 14.68±4.2 antral follicles (AFC). A significant correlation was observed between AMH and age (r=-0.34 *p*<.01), follicle stimulating hormone (FSH) serum levels (r=-0.32, *p*<.01), AFC (r=0.81, *p*<.00001), total dose of medication during ovarian stimulation (r=-0.28, *p*<.0003), and ongoing pregnancy rate (*p*<.05). Age was significantly correlated with FSH (r=0.46, *p*<.01), AFC (r=-0.34, *p*<.00001), total dose of medication during ovarian stimulation (r=0.43, *p*<.0003), and ongoing pregnancy rate (*p*<.04).

**Conclusion:**

Serum AMH and age are independent predictors of ovarian reserve and ovarian stimulation outcome in infertile women. Age and serum AMH level may be used to advise subfertile couples of their pregnancy prospects.

## INTRODUCTION

Female fecundity begins to decrease after women reach the age of 30 years, primarily as a result of decreases in the proportion of normal eggs available as a consequence of a continuous process of oocyte atresia. Although all women experience decreases in fecundity, it is difficult to predict the pace of reproductive decline in each individual. The age-related decline in fecundity is characterized by decreases in both egg quality and number, in addition to population-based changes in the expression of markers of ovarian activity, such as gradual increases in circulating FSH and decreases in circulating anti-Müllerian hormone (AMH) and inhibin B levels.

A classic report on the effects of female age on fertility found that the proportion of women off contraceptives unable to get pregnant increased steadily according to their age at the time of marriage: 6% at the ages of 20-24 years; 9% at the ages of 25-29 years; 15% at the ages of 30-34 years; 30% at the ages of 35-39 years; and 64% at the ages of 40-44 years (Menken *et al*., 1986). The age-associated decline in female fecundity and increase in risk of miscarriage have been largely ascribed to oocyte abnormalities. The meiotic spindle in the oocytes of older women frequently exhibits abnormalities in chromosome alignment and microtubular matrix composition (Battaglia *et al*., 1996). Higher rates of single chromatid abnormalities in oocytes (Angell, 1994), as well as aneuploidy in preimplantation embryos (Benadiva *et al*., 1996) and ongoing pregnancies, have been observed in older women. The higher rate of aneuploidy is a major cause of increased miscarriage and decreased live birth rates in women of advanced reproductive age.

Ovarian reserve evaluation has been the focus of substantial clinical research for several years (Navot *et al*., 1987; Hofmann *et al*., 1996; Toner *et al*., 1991; Frattarelli *et al*., 2000; Scott *&* Hofmann, 1995). Anti-Müllerian hormone (AMH), also known as Müllerian inhibiting substance, is a dimeric glycoprotein belonging to the transforming growth factor-b (TGF-b) superfamily, whose members include activins and inhibins, produced exclusively in the gonads, as shown more than two decades ago in animals (Vigier *et al*., 1984) and later in humans (Rey *et al*., 2003; di Clemente *et al*., 1992). In women, AMH is synthesized in the granulosa cells (GC) surrounding preantral and small antral follicles (Weenen *et al*., 2004; Durlinger *et al*., 2002). Despite the use of ultrasensitive assays, AMH is barely detectable in serum at birth. It reaches higher levels after puberty (Guibourdenche *et al*., 2003; Rajpert-De Meyts *et al*., 1999) and then declines with aging, until it becomes undetected again at menopause (La Marca *et al*., 2005). Although the physiological roles of AMH and the mechanisms involved in the regulation of the hormone are still poorly established, recent studies have singled AMH out as an attractive marker for assessing of ovarian activity. Baseline AMH, determined before stimulation (usually on day 3 of the cycle), was found to be a better measure of decreased ovarian reserve than classic parameters such as increases in follicle stimulating hormone (FSH) levels or decreases in inhibin B and antral follicle count (de Vet *et al*., 2002; Fanchin *et al*., 2003; 2005; Muttukrishna *et al*., 2005; Tremellen *et al*., 2005; Hazout, 2006). An inverse correlation was described between AMH and baseline FSH levels (Piltonen *et al*., 2005), in addition to age.

In assisted reproductive technology (ART), serum AMH has been described as a more reliable hormonal marker of ovarian response to controlled ovarian hyperstimulation (COH) with gonadotropins than baseline FSH, estradiol, inhibin B or female patient age (Anckaert *et al*., 2012; Hazout *et al*., 2004; Muttukrishna *et al*., 2004; Nardo *et al.,* 2009; Peñarrubia *et al*., 2005; Seifer *et al*., 2002). AMH has also been claimed to possess at least the same level of accuracy as the antral follicle count (AFC) as a predictor of poor (Broer *et al*., 2009) and excessive (Broer *et al*., 2011) response. In addition, high serum AMH levels before the start of COH have been associated with increased risk of ovarian hyperstimulation syndrome (OHSS) (Lee *et al*., 2008; Nelson *et al*., 2007). As with other ovarian reserve tests, AMH is not a good predictor of embryo quality or pregnancy in COS cycles, suggesting that AMH is a marker of quantitative rather than qualitative aspects of ovarian reserve (Rey *et al*., 2003; Anckaert *et al*., 2012; Broer *et al*., 2011; Nelson *et al*., 2007; Smeenk *et al*., 2007). However, age has been described as a good predictor of embryo quality (Scheffer *et al*., 2017a).

The aim of the present study was to investigate and compare the correlations of AMH and age with prognostic parameters and outcomes of assisted reproductive technology (ART) treatment.

## MATERIAL AND METHODS

### Subjects

This prospective study included 132 infertile women aged 24-48 years undergoing routine exploration during unstimulated cycles prior to the start of assisted reproductive technology (ART) treatments at our center from July 2015 to January 2017. Enrolled patients met the following inclusion criteria: i) both ovaries present; ii) no current or past diseases affecting the ovaries or gonadotropin/sex steroid secretion, clearance, or excretion; iii) no current hormone therapy; iv) adequate visualization of the ovaries on transvaginal ultrasound scans; and v) total number of small antral follicles (3-12 mm in diameter) between 1 and 32 in both ovaries. All patients signed an informed consent form prior to inclusion in the study.

### Protocol

The patients were given leuprolide acetate (Lupron, Abbott, France). The GnRH-agonist was initiated at a dose of 2.0 mg/day during the mid-luteal phase, overlapping with approximately five days of oral contraceptive pill (OCP) administration (Diane 35, Schering, Brazil). Pituitary down-regulation was monitored and patients with adequate pituitary desensitization were started on a recombinant FSH regime (Gonal-F; Merck-Serono Pharmaceuticals, Italy) and the dose of GnRH-agonist was reduced to 1.0 mg/day. FSH was started with dosages between 150 and 300 IU/day for four days with or without human menopausal gonadotropin (hMG) (Menopur; Ferring Pharmaceuticals, Germany) based on AFC and AMH. Thereafter, the dose of FSH was individually adjusted according to estradiol (E2) response and vaginal ultrasound findings.

When two follicles reached a size ≥ 16-18 mm, 250 mg of recombinant human Chorionic Gonadotropin (Ovidrel, Merck-Serono Pharmaceuticals, Italy) were administered and oocyte retrieval occurred 35 to 36 hours later.

Intracytoplasmic sperm Injection (ICSI) was routinely performed in all fertilization procedures as described in the literature (Palermo *et al.,* 1992). Fertilization was evident when two pronuclei were observed. Embryos were cultured until the day of transfer (day 3) in IVF Global^®^ media (Life Global, Canada) supplemented with 10% synthetic serum substitute (SSS) and graded according to the criteria described by Veeck (1999) and Hsu *et al*. (1999) before transfer.

The number of embryos to be transferred was defined based on the guidelines published by the Brazilian Federal Board of Medicine (CFM).

Luteal phase support was achieved with micronized P_4_, 600 mg/day, administered continuously by vaginal route, starting on the evening of ET.

Ongoing pregnancy (OP) was assessed as biochemical pregnancy (BQ) and subsequent observation of one or more gestational sacs. Miscarriage was defined as a clinically recognized pregnancy loss occurred before 20 weeks of gestation.

### Hormone Measurements and Ultrasound Scans

On day 3 of the cycle preceding COH, the female patients had blood samples harvested by venipuncture to have their serum AMH and FSH levels measured, and had their follicles measured by transvaginal ultrasound.

AMH and FSH serum levels were determined using an automated multi-analysis system with chemiluminescence detection (ACS-180; Bayer Diagnostics, Puteaux, France). Serum AMH levels were determined using a second-generation enzyme-linked immunosorbent assay. Intra- and inter- assay coefficients of variation (CV) were <6% and <10%, respectively, with lower detection limit at 0.13 ng/ mL and linearity up to 21 ng/mL for AMH.

For FSH testing, functional sensitivity was 0.1 mIU/mL, and the intra- and inter-assay CV were 3% and 5%, respectively.

A single operator blinded to the hormone assay results performed the ultrasound examinations using a 3.7-9.3 MHz multi-frequency transvaginal probe (RIC5-9H; General Electric Medical Systems, Paris, France). The objective of ultrasound examination was to evaluate the number and size of small antral follicles. Follicles with mean diameters of 3-12 mm (mean of two orthogonal diameters) in both ovaries were considered. To optimize the reliability of ovarian follicular assessment, the ultrasound scanner was equipped with a tissue harmonic imaging system (Thomas & Rubin, 1998), which yielded improved image resolution and adequate recognition of follicular borders. Intra-analysis CV for follicular and ovarian measurements were <5%, and their lower limit of detection was 0.1 mm. In an effort to evaluate the bulk of granulosa cells in both ovaries, we calculated the mean follicle diameter (cumulative follicle diameter divided by the number of follicles measuring 3-12 mm in diameter in both ovaries) and the largest follicle diameter.

### Ethical approval

Written informed consent was obtained from all participants before inclusion in the study. The Ethics Committee of the Brazilian Institute of Assisted Reproduction approved the study

### Statistical Analysis

Descriptive parameters and patient characteristics were reported as mean (SD) or median (range) values depending on variable distribution.

Student’s t-test and the Wilcoxon signed-rank test were performed for continuous variables; Fisher's exact test was performed for categorical variables; and Pearson's correlation coefficient was calculated.

*p*<0.05 was considered statistically significant.

## RESULTS

At the time of the study, the 132 patients included had a mean age of 35.7±3.84 years, a BMI of 22.30±1.78 kg/m^2^, and a length of infertility of 2.66±2.03 years. On cycle day 3, the mean serum AMH level was 2.84±1.57ng/mL. At baseline, the patients had 14.68±4.2 antral follicles.

**Figure 1:**
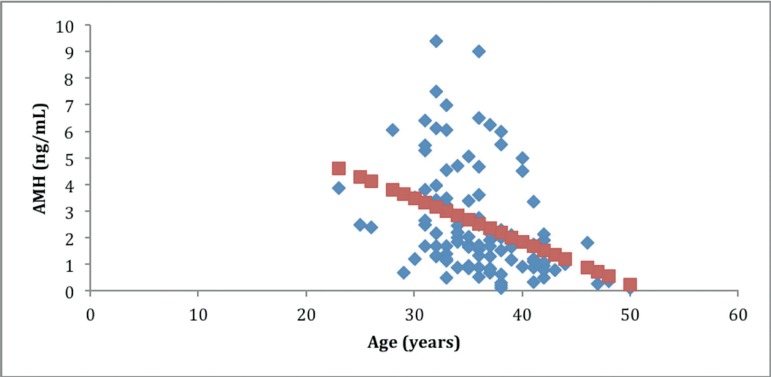
Correlation between anti-Müllerian hormone (AMH) and age

**Figure 2:**
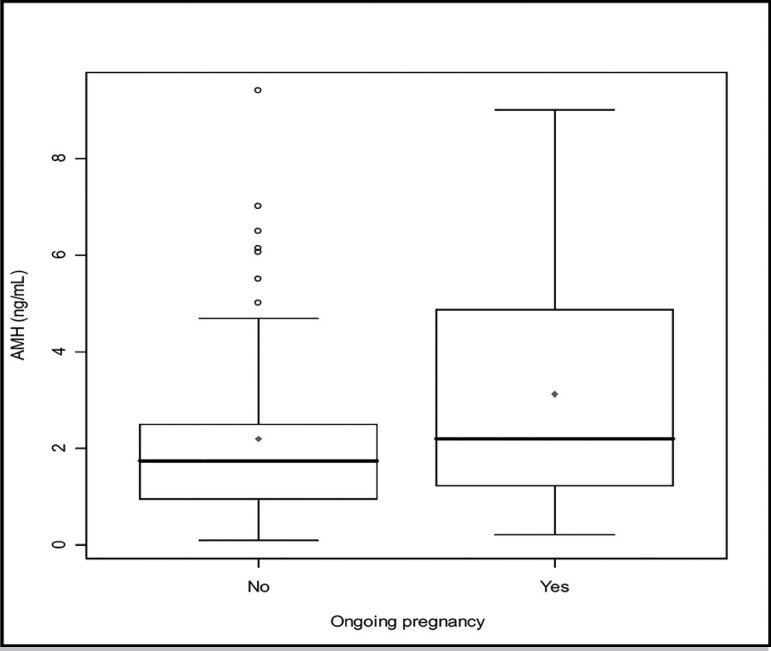
Comparison between serum anti-Müllerian hormone (AMH) levels of infertile patients with ongoing pregnancies and infertile patients without ongoing pregnancies. The box represents the interquartile range containing 50% of the values. The whiskers are lines that extend from the box to the highest and lowest values, excluding outliers. A line across the box indicates the median. *p*<.05, Student’s t-test

AMH was significantly correlated with age (r=-0.34 *p*<.01) (Figure 1), FSH (r=-0.32, *p*<.01), AFC (r=0.81, *p*<.00001), total dose of ovarian stimulation medication (r=-0.28, *p*<.0003), miscarriage rate (*p*<.02), and ongoing pregnancy rate (*p*<.05) (Figure 2).

**Figure 3:**
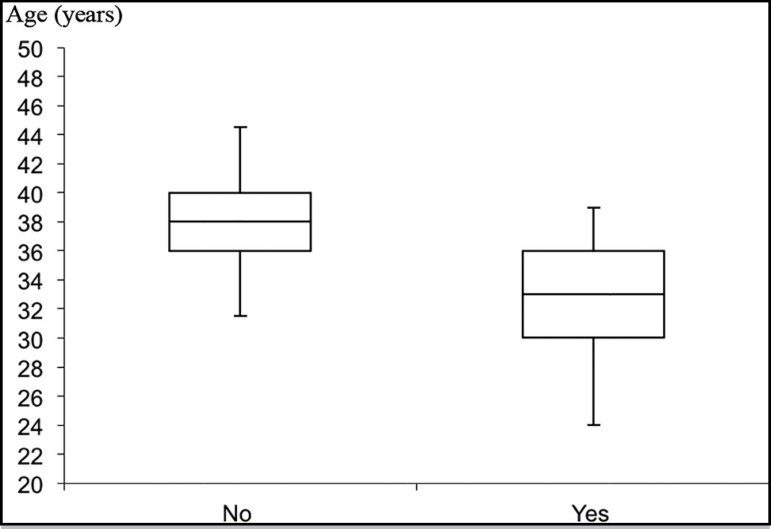
Comparison between the ages of infertile patients with ongoing pregnancies and infertile patients without ongoing pregnancies. The box represents the interquartile range containing 50% of the values. The whiskers are lines that extend from the box to the highest and lowest values, excluding outliers. A line across the box indicates the median. *p*<.05, Student’s t-test

Age was significantly correlated with FSH (r=0.46, *p*<.01), AFC (r=-0.34, *p*<.00001), total dose of ovarian stimulation medication (r=0.43, *p*<.0003), miscarriage rate (*p*<.03), an and ongoing pregnancy rate (*p*<.04) (Figure 3).

## DISCUSSION

This study demonstrated that serum AMH level and age were independent predictors of ovarian reserve and ovarian stimulation outcome in infertile women. Ovarian reserve is currently defined as the interplay between the quantity and quality of the follicles left in the ovary, and several proxy variables for pool size have been well described in the literature. Female reproductive aging is a process dominated by the gradual decline of oocyte quantity and quality (te Velde & Pearson, 2002). With increasing chronological age, female fecundity decreases (Broekmans *et al*., 2009). Progressive follicle decline is accompanied by notable changes in menstrual cycle regularity, with menopause as the final step in the ovarian aging process (Faddy *et al*., 1992; Hansen *et al*., 2008; Wallace & Kelsey, 2004).

Age has been used as a predictor of the number of harvested oocytes, number of metaphase II oocytes, and embryo quality. This marker of ovarian reserve is the single best predictor of reproductive outcome in women, and oocyte is the locus of reproductive aging in women. Whole chromosomal nondisjunction and precocious sister chromatid separation have been correlated to maternal aging. Disturbance in sister chromatid cohesion might be a causal mechanism predisposing to premature chromatid separation and subsequently to nondisjunction in female meiosis. In addition, the asymmetry of female meiosis division might favor nonrandom meiotic segregation of chromosomes and chromatids.

Oocyte aging leads to increased mitochondrial DNA damage and decreased oxidative phosphorylation and ATP production. Mitochondrial mutations in follicular cells surrounding the oocytes have been correlated with maternal age, suggesting that oxidative phosphorylation in the follicle is compromised (Smeenk *et al*., 2007). Anti-Müllerian hormone has been correlated with increased miscarriage rates. This finding is surprising, since decreased quantitative ovarian reserve is considered to be a reflection of advanced ovarian aging, a variable clearly associated with increased rates of fetal aneuploidy and miscarriage (Levi *et al*., 2001; Elter *et al.,* 2005; Lekamge *et al.,* 2007). The correlations described in the literature between AMH serum levels and pregnancy rates (PR) after reproductive therapies such as IVF were not conclusive. Some authors (Lekamge *et al.,* 2007; Peñarrubia *et al.,* 2005; Fiçicioglu *et al.,* 2006; Laven *et al*., 2004) were unable to find a correlation between baseline AMH levels and pregnancy rates, whereas others (Broer *et al*., 2011; Laven *et al*., 2004; Eldar-Geva *et al*., 2005) observed an association between higher baseline serum AMH levels and higher clinical PR. Similarly, a study described a positive correlation between embryo scores and serum AMH levels at the time of hCG administration (Silberstein *et al*., 2006), while another group of authors, in a more recent study (Lekamge *et al.,* 2007), found no correlation between serum AMH on day 3 of a control cycle and embryo morphology. A study reported a significant association between serum AMH measured on the first day of a COH cycle and treatment outcome (pregnancy) using a cutoff level for negative predictive value (Fréour *et al*., 2006). Data heterogeneity hampers further comparisons between published findings.

In our study, total consumption of gonadotropins was statistically correlated with AMH and age. The relationship between serum AMH levels and controlled ovarian stimulation outcome observed in this study is in agreement with previous studies on serum AMH levels. Serum AMH seems to reflect the follicular pool, and its production is independent of the gonadotropin-dependent indicators of ovarian reserve (Seifer *et al.*, 2002; van Rooij *et al.,* 2002; Scheffer, 2017b).

An ideal ovarian reserve test should be reproducible, with limited inter- and intra-cycle variability, and highly specific to minimize the risk of incorrectly categorizing women as having decreased ovarian reserve. No measure of ovarian reserve is perfect; however, AMH level and age have yielded good predictive value. More studies are needed to improve the accuracy and interpretation of the current ovarian reserve markers to clearly define cut-off levels for each marker and find other markers more strongly correlated with the number of ova retrieved, embryo quality, and clinical pregnancy rates. Determining the etiology of maternal aging on oocyte competence might improve patient care and fertility treatment outcomes.

## CONCLUSION

Serum AMH level and age are predictors of ovarian reserve and ovarian stimulation outcome in infertile women. Age and serum AMH levels might be used to advise subfertile couples of their pregnancy prospects. These markers should be deemed as an important element in the contemporary practice of reproductive medicine.
